# Sessile serrated lesions of the appendix: a contemporary review of the literature

**DOI:** 10.1186/s12876-026-05117-5

**Published:** 2026-07-17

**Authors:** Berkenye Csonka, Tamás Lantos, Anita Sejben

**Affiliations:** 1 Department of Pathology, Albert Szent-Györgyi Medical School, University of Szeged, Állomás u. 1., Szeged, 6725 Hungary; 2 Department of Medical Physics and Informatics, University of Szeged, Szeged, 6720 Hungary

**Keywords:** Appendix, Appendiceal neoplasm, Sessile serrated lesion, Sessile serrated lesion with dysplasia

## Abstract

**Introduction:**

Sessile serrated lesions (SSLs) of the appendix remain a controversial entity due to evolving definitions, diagnostic criteria, and nomenclature. Shifts in diagnostic thresholds - from multiple serrated crypts to a single unequivocally serrated and distorted crypt - have led to discrepancies in lesion classification and retrospective analyses. These controversies, compounded by inconsistent adherence to updated criteria, highlight the need for studies and reviews applying contemporary standards to accurately assess incidence and clinical significance.

**Methods:**

The review protocol was registered in PROSPERO (CRD420261299191). Systematic searches of PubMed, Web of Science, Scopus and Ovid were performed for studies published between 2007 and 2025. Eligible studies reporting clinicopathological features of appendiceal SSLs were included.

**Results:**

The reported prevalence of appendiceal SSLs is low (approximately 1.11–5.22‰), but increases with age and is markedly higher in patients with serrated polyposis syndrome. Most lesions are incidentally detected histologically, are often macroscopically inconspicuous, and show dysplasia in a minority of cases (~ 28%). Compared with colonic SSLs, appendiceal lesions are less common, show female predominance, and more frequently harbour *KRAS* rather than *BRAF* mutations, suggesting partially distinct molecular pathways. Although occasional co-occurrence with mucinous neoplasms and adenocarcinoma may suggest possible biological links, current evidence is insufficient to define a consistent progression pathway or establish their true malignant potential.

**Conclusions:**

Overall, the current literature is constrained by small sample sizes, heterogeneous study designs, inconsistent reporting, and substantial publication bias, particularly toward unusual or concurrent neoplastic findings. These limitations preclude definitive conclusions regarding the true prevalence, natural history, and malignant potential of appendiceal SSLs. Further large-scale, systematically characterised, and longitudinally followed cohorts with comprehensive molecular profiling are required to clarify their biological behaviour and clinical significance.

## Practitioner points

1. Evolving definitions of appendiceal sessile serrated lesions (SSLs) - particularly the shift to accepting a single unequivocally serrated crypt - have led to inconsistent classification across studies and in practice, underscoring the need for strict adherence to contemporary diagnostic criteria.
2. These lesions have a very low reported prevalence, are typically not visible macroscopically, and are most often identified incidentally on histology; while dysplasia occurs in a minority of cases, their true malignant potential remains unclear.
3. Appendiceal SSLs show distinct features compared with their colonic counterparts (e.g., female predominance and more frequent *KRAS* mutations), suggesting different molecular pathways, yet current evidence is insufficient to establish progression risk or inform specific surveillance or treatment strategies.

## Introduction

Sessile serrated lesions (SSLs) of the vermiform appendix have been a subject of ongoing debate within the field of pathology over recent decades. Currently, based on the 6^th^ edition of the World Health Organization (WHO) Classification of Tumours of the Digestive System, SSLs are defined as mucosal epithelial polyps, with a serrated architecture of the intestinal crypt lumen, the serrations and dilations extending to the bases of the crypts, which exhibit abnormal configurations, including L-shaped and inverted T-shaped forms. The presence of dysplasia should be explicitly indicated in the diagnosis, designated as SSL with dysplasia (SSL-D), dysplasia is further categorised as conventional adenoma-like, serrated, or traditional serrated adenoma (TSA)-like dysplasia [1]. 

Multiple changes in nomenclature regarding appendiceal SSLs have complicated long-term comparisons. The most recent revision occurred in 2019 with the publication of the 5TH edition of the WHO Classification, which no longer recommends the previously used term sessile serrated polyp/adenoma (SSP/A). Additionally, explicit definitions for appendiceal lesions were introduced, whereas the 4th edition provided only the term itself, requiring pathologists to rely on criteria established for colorectal lesions [2, 3]. Multiple publications have addressed the nomenclature change and expressed their positions on the issue. Before the introduction of the term SSL, pathologists used either “polyp” or “adenoma,” depending on whether greater emphasis was placed on morphological features or the lesion’s premalignant potential. However, SSLs often lack polypoid morphology, and traditional dysplastic features are not uniformly present either; unifying these terms into a more inclusive and descriptive term was justified [4]. Conversely, some researchers argue that omitting the term “adenoma” from the nomenclature may reduce clinicians’ perception of the lesion’s premalignant potential, possibly leading to less vigilant patient management and follow-up [5]. The presence of publications still using the old nomenclature may illustrate a reluctance on the part of the researchers to adhere to the term change.

Apart from the nominal modernisation, a minor change in the definition of the lesion has been implemented as well. The primary descriptors of the lesions remain unchanged; however, in the 4TH edition of the WHO Classification, researchers specified that a minimum of three intestinal crypts exhibiting serrated architecture, along with the characteristic basal crypt features, are required to meet the diagnostic criteria. The updated 5^th^ and 6^th^ editions, however, deem the presence of a singular, but unequivocally description-accurate crypt satisfactory to diagnose an SSL [1–4, 6]. A study of proximal colonic SSLs re-evaluated hyperplastic polyps (HPs) diagnosed prior to the 5TH edition of the WHO Classification and found that, based on current criteria, 13.3% required reclassification as SSLs [6]. Apparent differences in the reported incidence of SSP/As versus SSLs may reflect changes in diagnostic criteria, and interpreting these trends without accounting for such shifts could lead to an exaggerated perception of a recent increase in SSL cases.

The variation in diagnostic criteria underscores that any review of appendiceal SSLs should include only studies applying the updated terminology and standards. Currently, there is a notable lack of such reviews, with only one existing example addressing serrated lesions of the appendix under the new nomenclature; however, most of its references predate the terminology change and combine results from different diagnostic eras [7]. Our aim is to address this gap by providing a review that ensures consistency in diagnostic criteria and terminology across included studies.

## Methods

Our systematic review was conducted in accordance with the Preferred Reporting Items for Systematic Reviews and Meta-Analyses (PRISMA) 2020 guidelines, and the study protocol was registered with the National Institute for Health Research Prospective Register of Systematic Reviews (PROSPERO) database [CRD420261299191].

### Search strategy

A comprehensive, systematic literature search was conducted independently by three reviewers (B.Cs., A.S., T.L.) using four electronic databases: PubMed, Web of Science, Scopus, and Ovid. The search was completed on 26 January 2026. A standardised search strategy was applied across all databases, using the following formula: ((appendix) OR (appendiceal)) AND ((sessile serrated lesion) OR (SSL)).

### Selection and eligibility criteria

Based on the search strategy outlined above, original research studies, case reports, and abstracts were considered eligible for inclusion, whereas review articles were strictly excluded. Studies published in languages other than English were also excluded. Eligible studies were required to report on SSLs of the appendix and to comply with the current gastrointestinal neoplasm nomenclature guidelines published by the WHO in 2019 and 2025 [1, 2]. Duplicate records were identified and removed by the reviewers (B.Cs., A.S., T.L.) using predefined criteria, including article title, publication year, and author identifiers. After duplicate removal, three independent reviewers (B.Cs., A.S., T.L.) systematically assessed all remaining records through title, abstract, and full-text screening stages.

### Data extraction

Three independent reviewers (B.Cs., A.S., T.L.) systematically extracted data from eligible studies using a standardised data collection form. Information retrieved from the full texts and supplementary materials included: article title, first author, year of publication, Digital Object Identifier (DOI), study type, definition of SSL used in the publication, number of patients, male-to-female ratio, mean patient age at diagnosis, anatomical location of the appendiceal SSL, largest tumour diameter (mm), clinical presentation, preliminary clinical diagnosis, presence of dysplasia, number of pathologists evaluating histology, associations with colorectal dysplasia or carcinoma, immunohistochemistry (IHC) results (if performed), molecular genetic analysis results (if performed), mean and median follow-up durations, and any additional remarks considered relevant by the reviewers.

## Results 

### Screening results

**Fig. 1:**
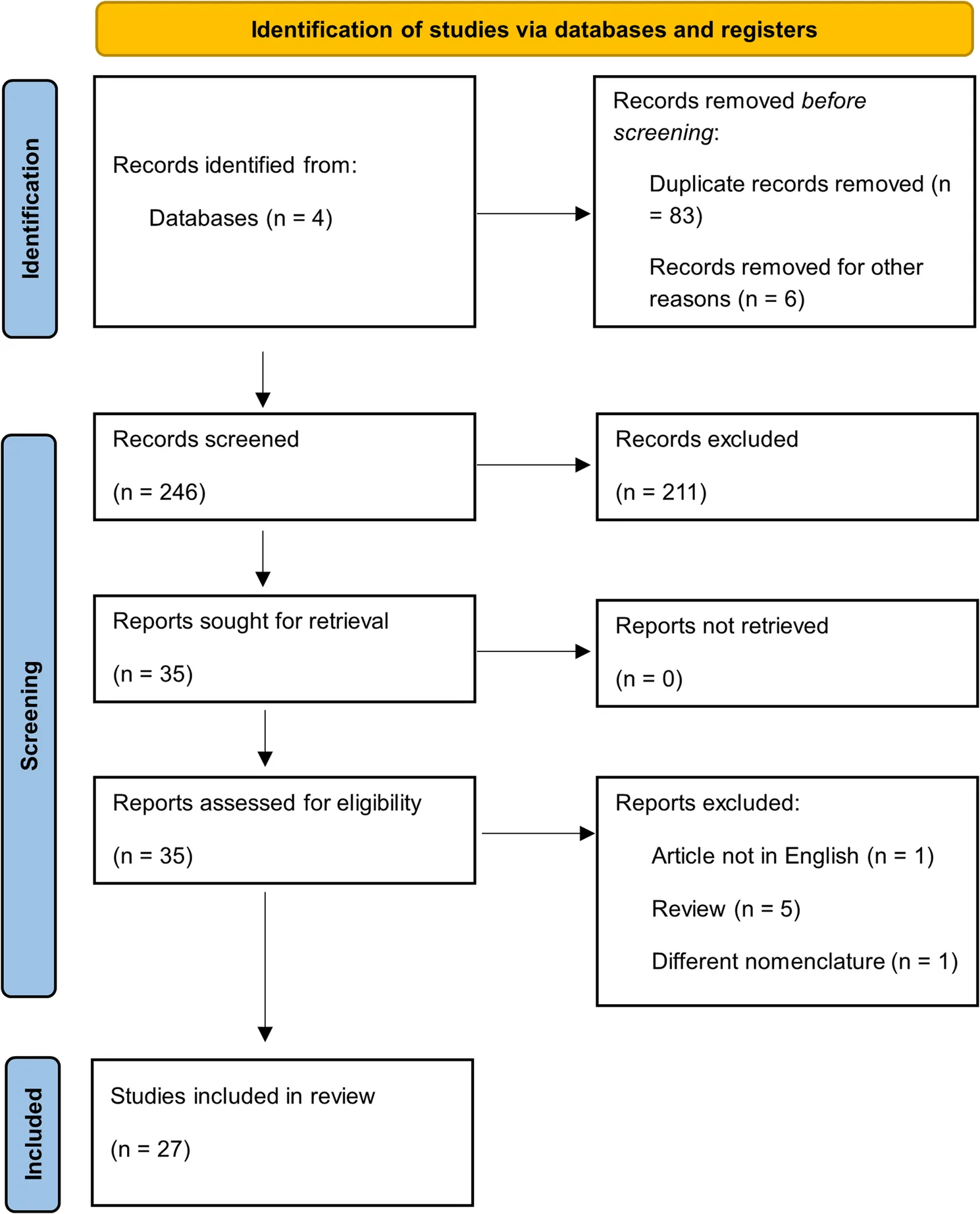
PRISMA flow diagram showing the study selection process for the systematic review of appendiceal SSLs

The systematic search of PubMed, Web of Science, Scopus and Ovid identified a total of 335 potential articles, including 83 duplicates. After removal of duplicates and an additional six articles that were book chapters, the remaining 246 records were screened following the inclusion and exclusion criteria through abstract screening, and 35 articles remained. All 35 articles were retrievable and were assessed for eligibility via full-text review. Additional 8 publications were excluded as they did not meet the inclusion criteria. Ultimately, 27 studies were included in the final analysis. Case reports (*n* = 14; 51.85%) dominate the included publications, followed by single-centre, retrospective studies (*n* = 10; 37.03%). In addition, the dataset included two case series (7.41%) and one single-centre prospective study (3.70%). The PRISMA flow diagram is shown in Fig. 1, and a summary of all extracted data is provided in Table 1.

**Table 1:** Summary of data presented in the studies included in this review

1^st^ author	Year of publication	Definition of SSL given/WHO	No. of patients	Male: female ratio	Mean age (years)	Localisation of SSL	Largest diameter (mm)	Clinical manifestation	Presence of dysplasia	Association with colorectal dysplasia	Association with CRC	IHC carried out	Molecular genetic analysis	Mean follow-up (months)	Country
Kinoshita	2014	✓	1	0:1	72	P	28	Asymp.	∅	NA	1/1	✓	∅	9	Japan
Koç	2020	✓	1	0:1	86	NA	90	AAp.	NA	NA	NA	∅	∅	33	Turkey
Munari	2021	∅	18	NA	NA	NA	NA	NA	3/18	NA	NA	✓	✓	NA	Italy
Dargent	2021	✓	1	0:1	15	M	8	AAp.	∅	∅	∅	✓	✓	36	Belgium
Chen	2021	∅	4	2:2	59	P	20	NA	NA	NA	NA	∅	∅	3	China
Lu	2022	✓	64	28:36	NA^1^	NA^2^	50	AAp.	32/64	NA	NA	∅	∅	NA	China
Takada	2022	∅	1	1:0	75	P	NA	Asymp.	∅	NA	NA	∅	∅	NA	Japan
Chezar	2022	✓	32	12:20	62.5	NA	NA	NA	NA	NA	NA	✓	✓	partial^3^	Canada
Morikawa	2022	∅	1	1:0	69	P	NA	NA	NA	NA	NA	∅	∅	NA	Japan
Toledo	2023	✓	15	NA	NA	NA	NA	Asymp.	1/15	15/15	NA	∅	∅	NA	The Netherlands
Kamitami	2023	∅	1	1:0	55	P	8	NA	1/1	NA	NA	∅	∅	NA	Japan
Jolly	2023	∅	6	NA	NA^4^	NA	NA	AAp.	NA	NA	1/6	∅	∅	NA	Australis
Justiniano	2024	∅	20	NA	NA	NA	NA	NA	NA	NA	N0	∅	∅	8	USA
Ahmed	2024	∅	1	0:1	68	P	10	Asymp.	∅	∅	N0	∅	∅	NA	Canada
Yao	2024	✓	1	0:1	54	M	NA	Asymp.	NA	1/1	∅	∅	∅	NA	China
Yao	2025	∅	1	0:1	36	P	15	Asymp.	NA	NA	NA	∅	∅	NA	China
Yang	2025	✓	1	0:1	55	P	NA	Constipation	NA	NA	NA	∅	∅	NA	China
Zhang	2025	✓	1	0:1	53	P	NA	Asymp.	1/1	NA	NA	∅	∅	4	China
Mahadik	2025	✓	23	7:17	68	P	NA	AAp.	2/23	NA	1/23	∅	✓	26	USA
Harris	2025	∅	1	0:1	47	P	8	Asymp.	∅	NA	NA	∅	∅	NA	USA
Gutiérrez-Rios	2025	∅	2	NA	NA	P	15	Asymp.	NA	NA	NA	∅	∅	24	Spain
Dai	2025	∅	1	1:0	67	M	12	AAp.	1/1	NA	NA	∅	∅	NA	China
Bauer	2025	✓	6	2:4	67	NA	NA	NA	1/6	NA	NA	∅	✓	NA	USA
Michael	2025	∅	1	0:1	78	NA	NA	Chr. RLQ pain	1/1	NA	NA	∅	∅	NA	USA
Csonka	2025	✓	19	10:9	62	NA	NA	AAp.	NA	1/19	10/19	∅	∅	30.3	Hungary
Wurtz	2025	✓	1	01	64	P	P	Asymp.	NA	∅	∅	∅	∅	6	USA
Khalid	2025	✓	7	0:7	72.3	NA	NA	Asymp.	∅	NA	NA	∅	∅	NA	UK

*Abbreviations*: *AAp.* acute appendicitis, *Asymp.* asymptomatic, *Chr.* chronic, *IHC* immunohistochemistry, *RLQ* right lower quadrant, *SSL* sessile serrated lesion, *WHO* World Health Organization

^1^ A total of 71.88% of patients were aged over 60 years

^2^ Complete appendiceal involvement was observed in 17.19% of lesions

^3^ Follow-up of 36–72 months was performed in co-occurring pT4 LAMN cases (*n* = 5)

^4^ The cohort consisted exclusively of patients aged >40 years

**Fig. 2:**
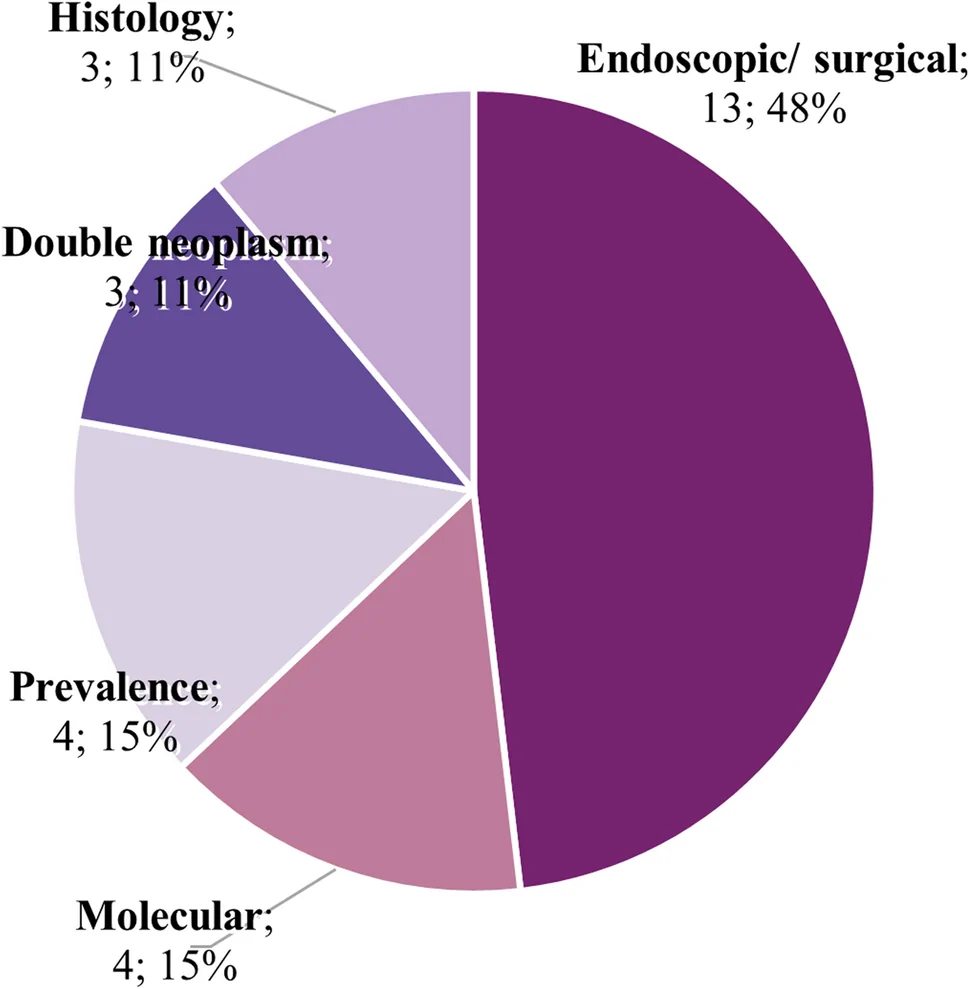
Results of the screening assay, highlighting the main focus of the articles

The focus of these studies, beyond reporting on appendiceal SSLs, was highly heterogeneous. The majority primarily addressed endoscopic detection, advanced visualisation techniques, and endoscopic therapeutic approaches for appendiceal SSLs [8–20]. Four studies reported on the prevalence of appendiceal neoplasms, including SSLs, across diverse clinical contexts, such as appendectomy specimens, patients with serrated polyposis syndrome (SPS), and surgical specimens from gynaecological malignancies that included appendectomy [21–24]. Four studies aimed to perform molecular characterisation of SSLs using IHC and/or molecular genetic analyses [25–28]. Three studies specifically investigated the co-occurrence of appendiceal SSLs with other primary appendiceal malignancies [29–31]. Finally, 3 studies focused on the micromorphological features of SSLs, including the re-evaluation of histological sections diagnosed prior to 2019, as well as potential differential diagnostic pitfalls in distinguishing SSLs from low-grade appendiceal mucinous neoplasms (LAMN) [32–34]. Fig. 2 illustrates the primary focus of the extracted articles.

### Population data

**Fig. 3:**
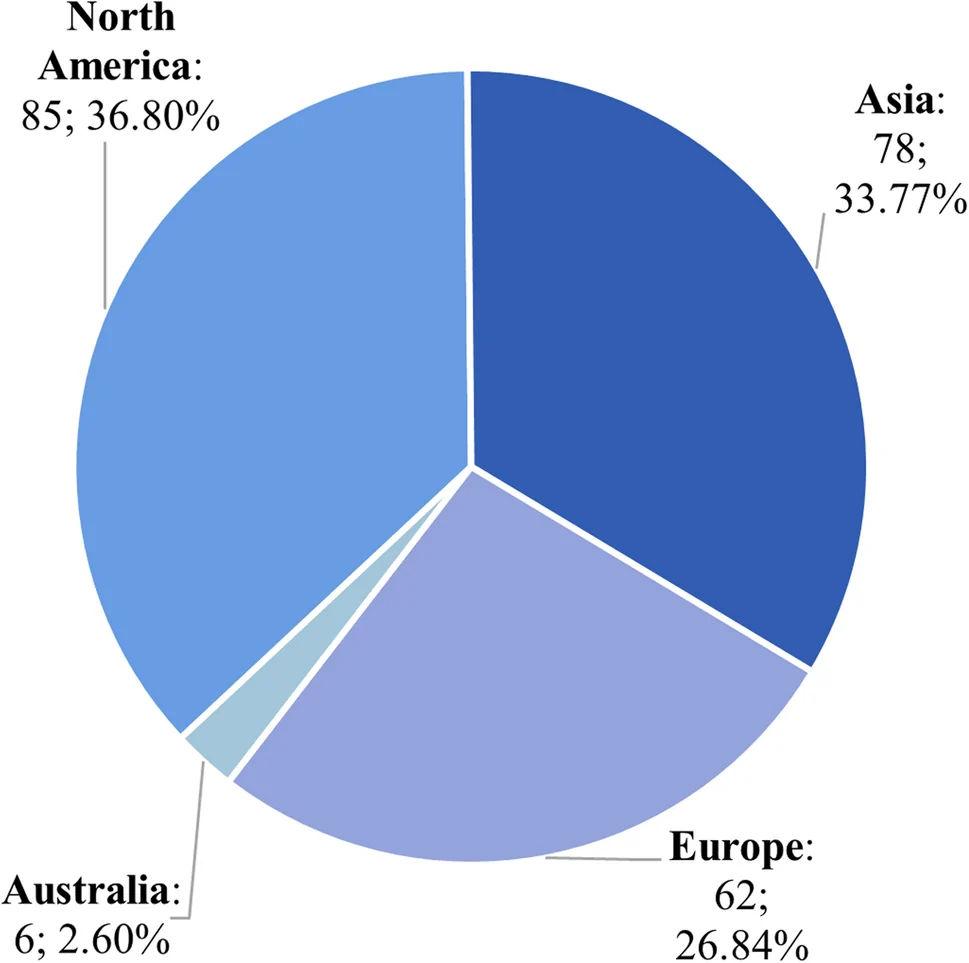
Geographical distribution of patients with appendiceal (SSLs)

The studies were conducted across 12 countries spanning 4 continents. The highest number originated from China (*n* = 7), followed by the United States (*n* = 6) [9, 10, 12, 13, 17–20, 25, 30, 31, 33, 34]. On average, 8.6 cases of appendiceal SSLs were reported per study (range: 1–64), yielding a total of 231 cases. Some studies also included patients with other appendiceal neoplasms; however, these cases were excluded from the present analysis [21–26, 28, 32–34]. The majority of patients were from North America (*n* = 85; 36.80%), followed by Asia (*n* = 78; 33.77%). European studies contributed data on 62 patients (26.84%), while a single study conducted in Australia accounted for an additional 6 patients (2.60%). Individual-level data were available or obtained from corresponding authors upon request in 20 studies (74.07%), comprising 83 cases (35.93%). The remaining data were reported in aggregated form; in some instances, partial individual case information could be extracted or incorporated into the original descriptive statistics. Figure 3 illustrates the geographical distribution of patients with appendiceal SSLs.

**Fig. 4:**
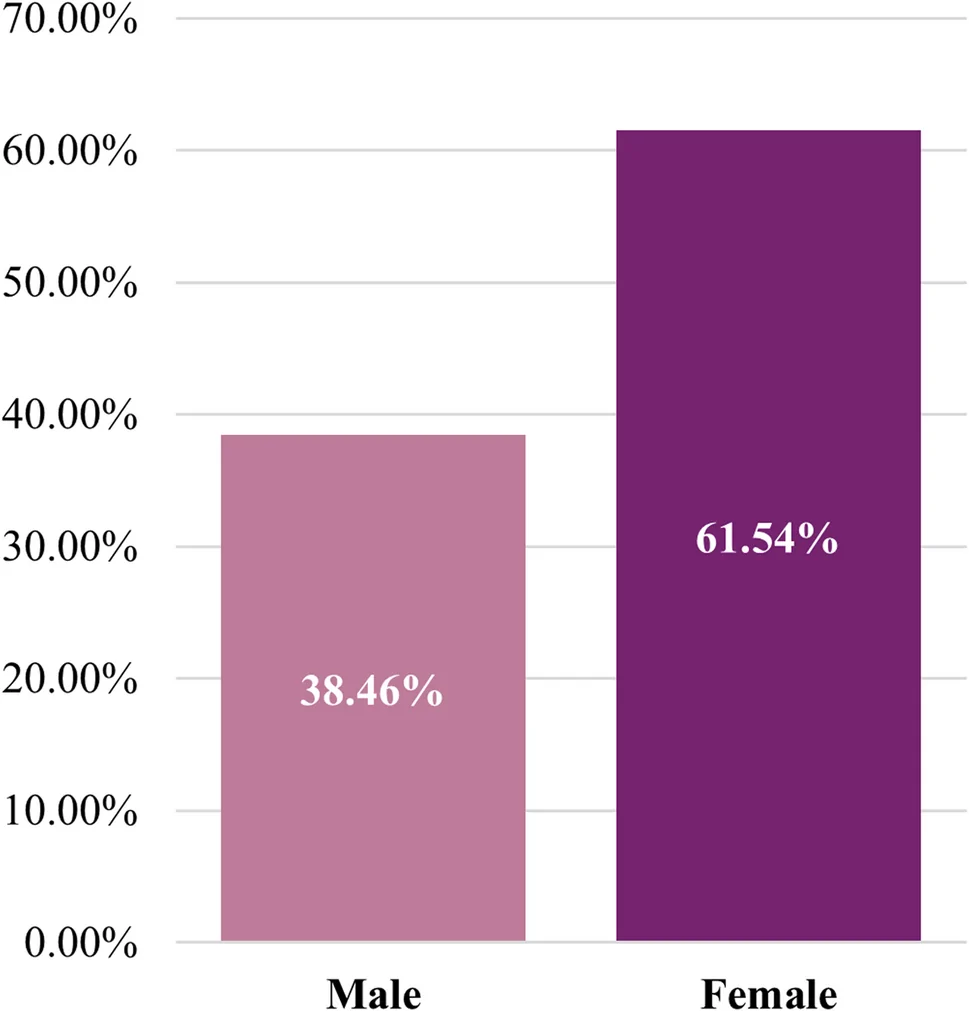
Sex distribution of patients with appendiceal SSLs, showing a female predominance

Sex was reported in 169 cases (73.16%). A female predominance was observed (*n* = 104; 61.54%), while males accounted for 38.46% (*n* = 65). The male-to-female ratio is shown in Fig. 4.

**Fig. 5:**
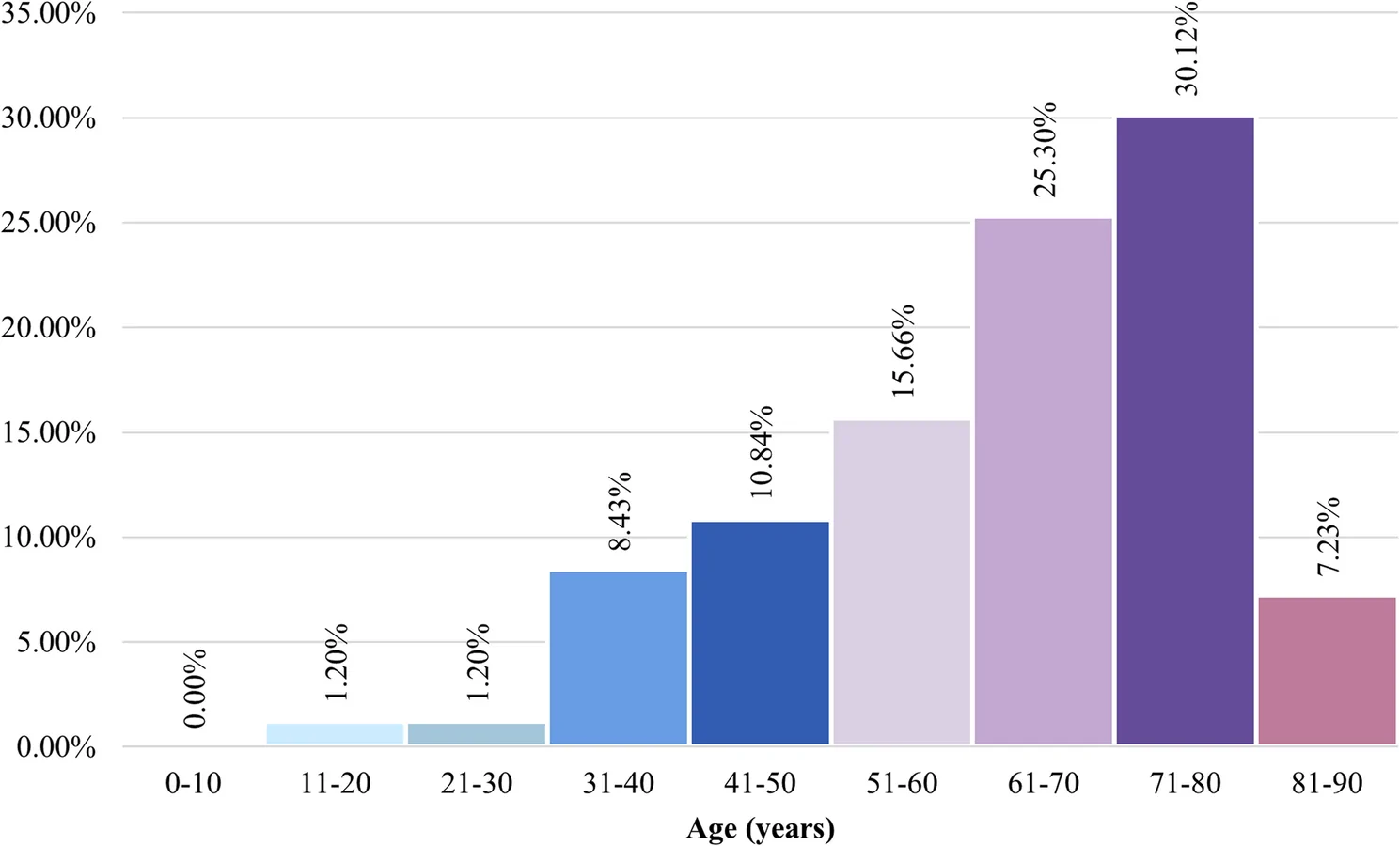
Age distribution of patients with appendiceal SSLs

Patient age was reported in 83 cases (35.93%). The mean age at diagnosis of appendiceal SSLs was 62.9 years, with a range of 15–90 years. 17 patients (20.48%) were younger than 50 years. More than half of the patients fell within the 61–80 years age group. The detailed age distribution is presented in Fig. 5.

### Clinicopathologic features of appendiceal SSL

**Fig. 6:**
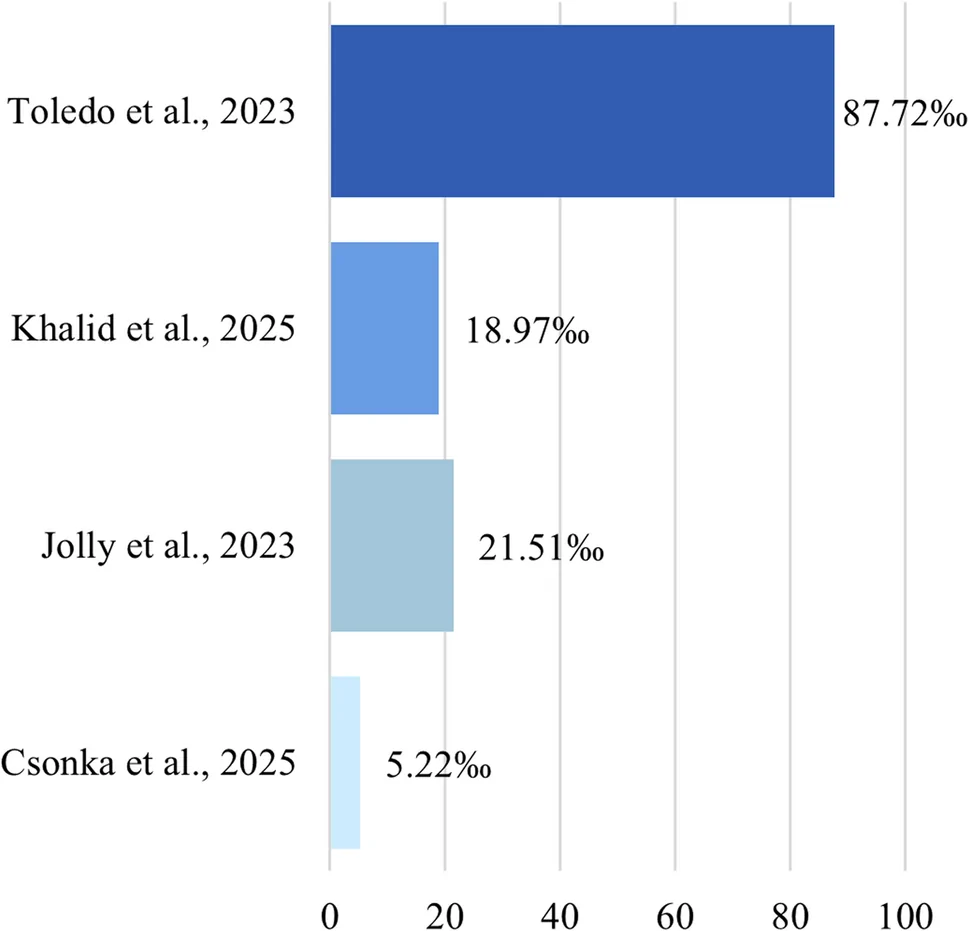
Prevalence of appendiceal SSL in different clinical settings

The 4 available studies reporting on the prevalence of appendiceal SSLs across different clinical settings demonstrate considerable variability in their findings. Csonka et al. evaluated a general population of patients undergoing appendectomy for appendicitis and reported a prevalence of 5.22‰ (19/3640) [21]. In contrast, Jolly et al. restricted their cohort to patients over 40 years of age with appendicitis, observing a higher prevalence of 21.51‰ (6/279) [22]. In a distinct high-risk population, Toledo et al. prospectively followed patients with SPS, among whom appendiceal SSLs were identified at a markedly increased prevalence of 87.72‰ (15/171) [24]. Finally, Khalid et al. reported a prevalence of 18.97‰ (7/369) in a cohort of patients undergoing surgery for gynaecological malignancies [23]. Taken together, these data highlight substantial heterogeneity in reported prevalence rates, likely reflecting differences in patient selection, underlying risk profiles, and study design. The prevalence of SSLs in each study is illustrated in Fig. 6.

**Fig. 7:**
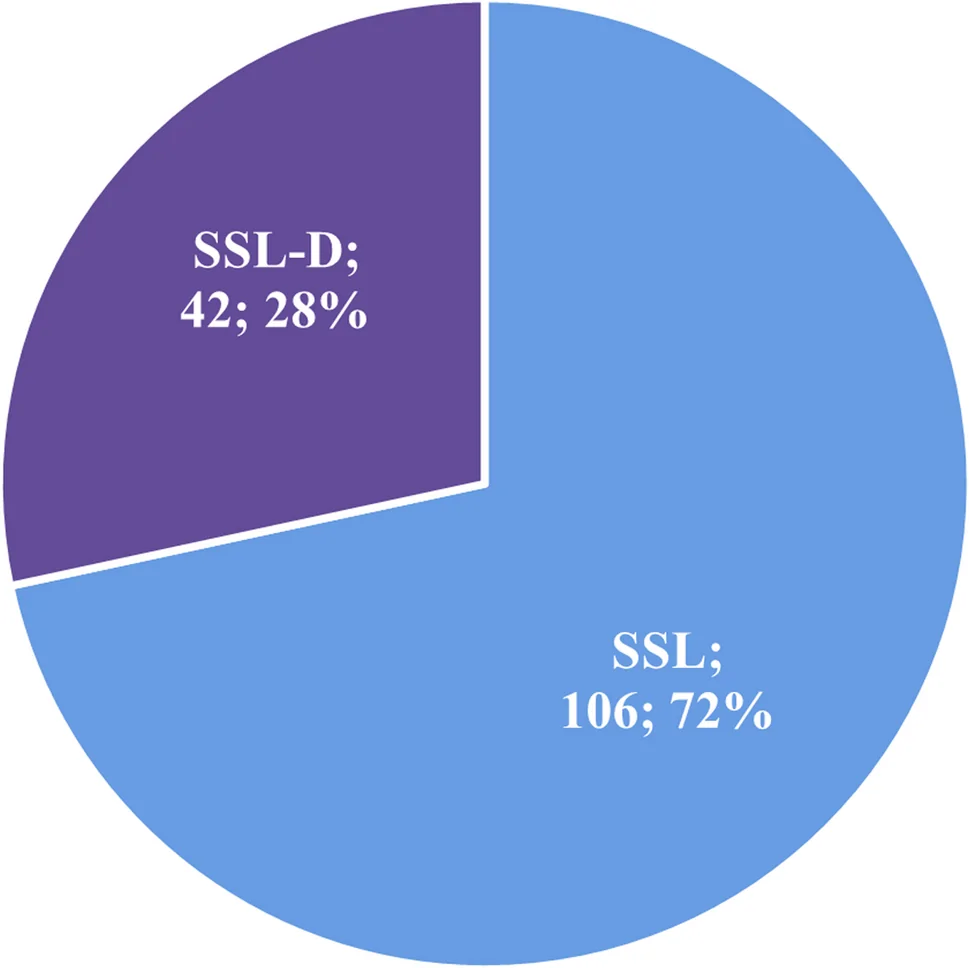
Proportion of SSL-D in the examined populations. Abbreviations: SSL - sessile serrated lesion; SSL-D - sessile serrated lesion with dysplasia

Most appendiceal SSLs were reported to be located either proximally or at the appendiceal orifice (*n* = 36; 91.31%). Notably, the exact lesion location was specified in only 16.88% of cases (*n* = 39), all within endoscopy-focused case reports. Data on the presence of dysplasia were available for 148 lesions (64.07%). The majority of lesions (*n* = 106; 71.62%) showed no dysplastic regions, whereas 42 lesions (28.38%) contained dysplasia. Among the three cases in which dysplasia was graded, one exhibited low-grade dysplasia, and two demonstrated high-grade dysplasia. The distribution of SSL and SSL-D in the examined studies is presented in Fig. 7.

Lesion size was reported in only 10.39% of cases (*n* = 24). Based on these limited data, the mean lesion diameter was 26.42 mm (range: 8–90 mm). However, this likely reflects a reporting bias, as many lesions were probably macroscopically inconspicuous, and therefore not measured. Furthermore, five studies, comprising a total of 80 cases [25, 26, 28, 32, 34], were based on previously grossed and histologically processed specimens, which likely precluded accurate assessment or reporting of macroscopic lesion size. Overall, macroscopic or preoperative detection of appendiceal lesions was documented in 9.96% of cases (*n* = 23). Among these, lesions were identified endoscopically in 18 cases, detected on preoperative imaging in 2 cases, and described as grossly visible by the surgeon or pathologist in three cases. Table 2 summarises the different pathological features of the cases.

**Table 2:** Localisation, presence of dysplasia and the largest diameter of appendiceal SSLs

	Reported (*n*)	Reported (%)		*n*	%
Localisation	39	16.88%	**Proximal**	36	91.31%
			**Middle**	3	7.69%
			**Distal**	0	0%
Presence of dysplasia	148	64.07%	**SSL**	106	71.62%
			**SSL-D**	42	28.38%

	Reported (*n*)	Reported (%)	Mean (mm)	Range (mm)	
Largest diameter of lesion	24	10.39%	26.42	8-90	

*Abbreviations*: *SSL* sessile serrated lesion, *SSL-D* sessile serrated lesion with dysplasia

### Clinical presentation and relevant patient history

**Fig. 8:**
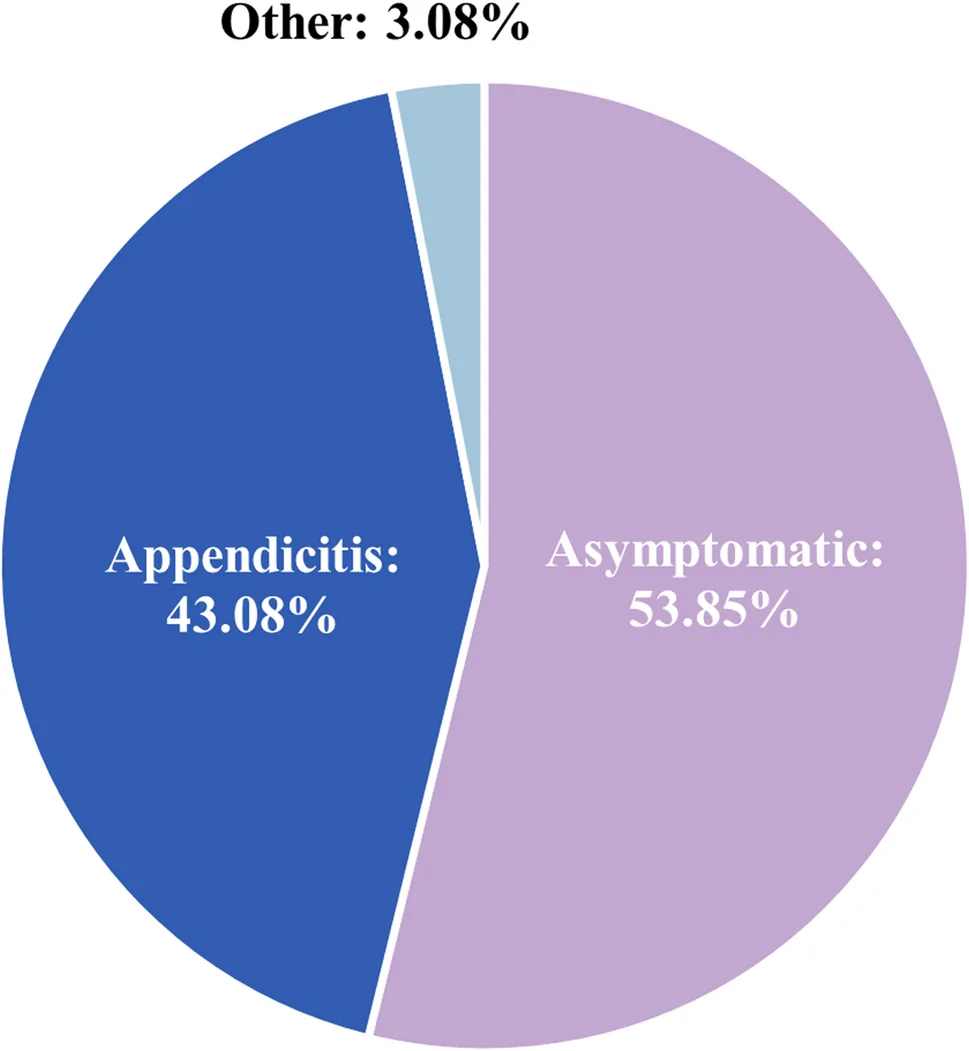
Clinical presentation of patients diagnosed with appendiceal SSL

The initial clinical presentation was documented in 65 cases (28.14%). In the majority of these, the appendiceal lesion itself was asymptomatic (*n* = 35; 53.85%). Among these asymptomatic cases, two lesions were incidentally detected during routine screening colonoscopy [8, 31], in one patient due to a positive family history of colorectal cancer [19], and in another through a positive stool immunochemical test [16]. A distinct asymptomatic subgroup of 15 patients consisted of individuals with previously diagnosed SPS who were prospectively evaluated [24]. Conversely, a substantial portion of patients (*n* = 28; 43.08%) presented with symptoms typical of acute appendicitis, most commonly acute right lower quadrant abdominal pain. Two additional cases presented with chronic right lower quadrant pain [30] or chronic constipation [17]. Fig. 8 illustrates the ratio of different clinical presentations in the context of appendiceal SSL.

In several cases, notable patient histories were reported. Three patients were diagnosed with appendiceal SSL involving the appendiceal stump. In one case, a growth was identified during grossing of a recently resected appendix, prompting a follow-up colonoscopy that revealed a residual polypoid lesion at the appendiceal orifice [15]. Two additional patients developed SSL in the appendiceal stump decades (20–37 years) after initial appendectomy for appendicitis [14, 20]. Three cases involved patients with a prior history of appendiceal orifice SSL. Two patients had longstanding lesions spanning 5–12 years, sampled repeatedly, SSL was confirmed on each occasion [14, 31]. Another patient experienced recurrence during follow-up after endoscopic mucosal resection (EMR) and underwent repeat EMR [13]. 

### Therapy, follow-up and patient outcomes

**Fig. 9:**
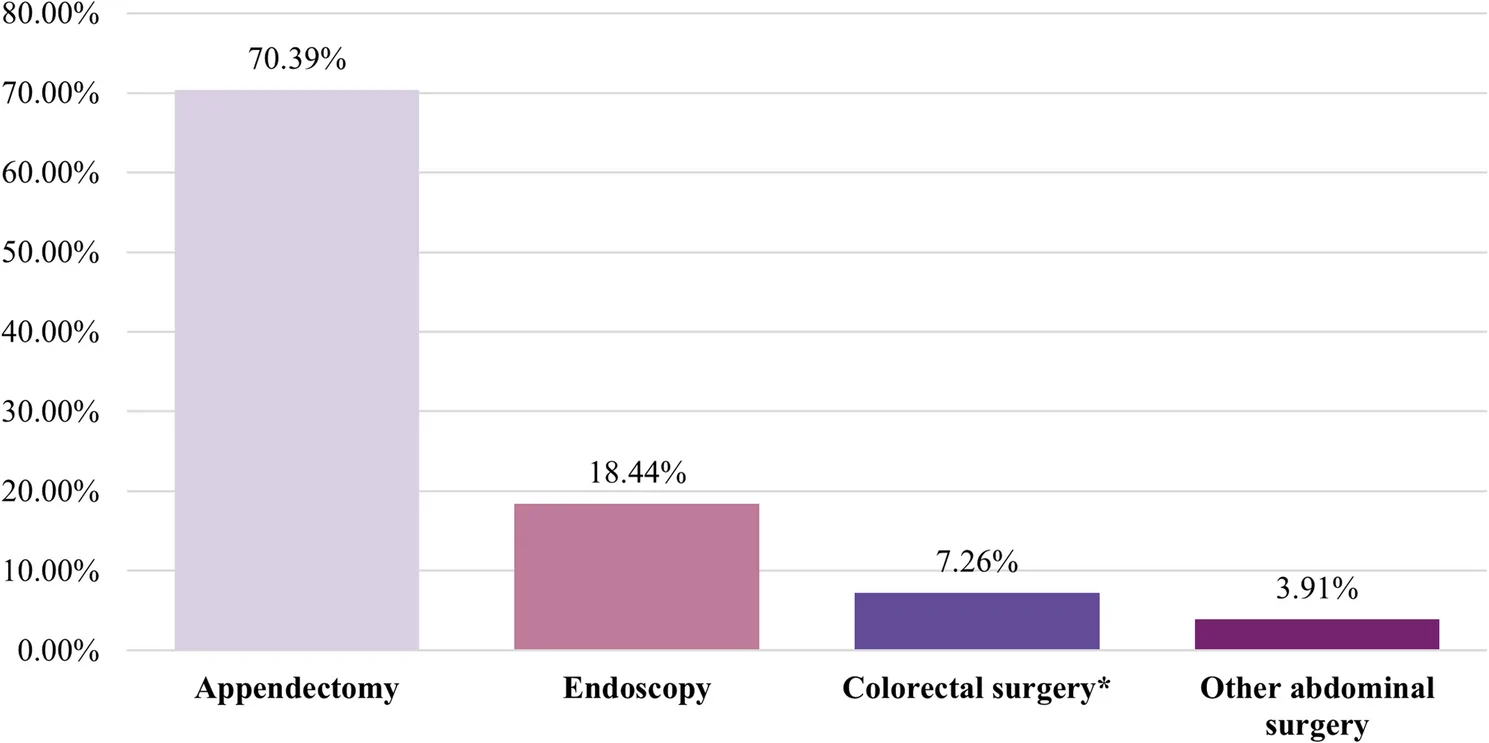
Distribution of different treatment options among appendiceal SSL cases. * Including subtotal colectomy, total colectomy, right hemicolectomy and ileocecal resection

Therapeutic modality was reported in 179 cases (77.49%). The majority of appendiceal SSLs were managed with appendectomy alone (*n* = 126; 70.39%). One case explicitly described an open appendectomy [32], whereas the remaining cases are presumed to have been treated laparoscopically. Endoscopic removal was performed in 33 cases (18.44%), primarily for proximally located polyps. Larger colorectal resections were required in 13 cases (7.26%) due to involvement of the ileocecal region or concomitant colorectal neoplasms. Additionally, one study selectively included seven patients (3.91%) with gynaecological malignancies in whom appendices were removed alongside the primary surgery [23]. Fig. 9 illustrates the distribution of therapeutic approaches across the reported cases.

Follow-up data were available for 35 cases (15.15%). The mean follow-up duration was 26.1 months (range: 0–101 months). During this period, one lesion recurrence was reported, as described above. Four patients died during follow-up, occurring 5–33 months after SSL diagnosis at ages 55, 86, and 90; the age of the fourth patient was not reported. One of these cases involved co-occurring SSL and appendiceal signet ring cell adenocarcinoma; the patient died 22 months after diagnosis due to progression to stage IV carcinoma [34]. For the remaining 3 cases, the cause of death and any potential relation to SSL were not specified [21, 32]. 

### Association with other appendiceal neoplasms

A total of 5 studies, comprising 23 cases, have reported co-occurrence of different appendiceal neoplasms [25, 26, 29, 31, 34]. Most SSL cases were associated with LAMN (*n* = 13; 5.63%). The mean age of patients with SSL and LAMN was 67.54 years (range: 46–82 years), and 46.15% of the cases were female (male-to-female ratio: 7:6). Adenocarcinomas and goblet cell adenocarcinomas were the second most common histologic findings, each reported in 4 cases (1.73%). Of the 4 adenocarcinoma cases, 2 were mucinous subtypes. The mean age of patients with SSL and adenocarcinoma was 64.5 years (range: 46–72 years). 75% of the patients were female (male-to-female ratio: 1:3). The mean age of patients with SSL and goblet cell adenocarcinoma was 76 years (range: 73–79). Similar to concurring adenocarcinomas, 75% of the patients were female (male-to-female ratio: 1:3). There was one (0.3%) occurrence of both a neuroendocrine tumour (NET) and signet ring cell adenocarcinoma. The NET harbouring appendix belonged to a 64-year-old female patient. There was no individual data available regarding sex and age in the case of the co-occurring SSL and signet ring cell adenocarcinoma. Table 3 summarises the clinicopathological features of these concurrent appendiceal neoplastic cases.

**Table 3:** Demographic characteristics of co-occurring neoplasms in the appendix

Concurrent neoplasm	Sex	Age	Publication
LAMN	M	75	Chezar and Minoo, 2022 [26]
LAMN	M	82	Chezar and Minoo, 2022 [26]
LAMN	F	55	Chezar and Minoo, 2022 [26]
LAMN	M	76	Chezar and Minoo, 2022 [26]
LAMN	F	60	Chezar and Minoo, 2022 [26]
LAMN	F	46	Chezar and Minoo, 2022 [26]
LAMN	M	66	Chezar and Minoo, 2022 [26]
LAMN	F	61	Chezar and Minoo, 2022 [26]
LAMN	M	51	Chezar and Minoo, 2022 [26]
LAMN	M	78	Chezar and Minoo, 2022 [26]
LAMN	M	76	Chezar and Minoo, 2022 [26]
LAMN	F	79	Chezar and Minoo, 2022 [26]
LAMN	F	73	Bauer et al., 2025 [25]
Goblet cell adenocarcinoma	F	78	Bauer et al., 2025 [25]
Goblet cell adenocarcinoma	F	73	Bauer et al., 2025 [25]
Goblet cell adenocarcinoma	M	74	Bauer et al., 2025 [25]
Goblet cell adenocarcinoma	F	79	Bauer et al., 2025 [25]
Adenocarcinoma	M	62	Bauer et al., 2025 [25]
Adenocarcinoma	F	46	Bauer et al., 2025 [25]
Mucinous adenocarcinoma	F	78	Bauer et al., 2025 [25]
Mucinous adenocarcinoma	F	72	Kinoshita et al., 2014 [29]
NET	F	64	Wurtz et al., 2025
Signet ring cell adenocarcinoma	NA	NA	Mahadik et al., 2025 [34]

*Abbreviations*: *LAMN* low-grade appendiceal mucinous neoplasm, *NET* neuroendocrine tumour

### Association with colorectal dysplasia and cancer

Concurrent or anamnestic colorectal neoplasms were mentioned in 31 cases (13.42%), in relation to 27 patients. Of these, 18 cases (60.00%) were benign neoplasms or SPS, and 12 cases (40.00%) were malignant colorectal neoplasms [18, 21, 24, 29]. 

Beyond the 15 cases of SPS reported by Toledo et al., the identification of an appendiceal SSL in a 15-year-old patient raises the possibility of an underlying, yet undiagnosed SPS [24, 27]. Furthermore, Csonka et al. describe a patient with concomitant colonic HP and tubular adenoma in addition to an appendiceal SSL. Finally, Yao et al. report a case of a confluent cecal and appendiceal SSL, raising questions regarding the precise origin of the appendiceal lesion [18, 21]. 

Twelve cases of concurrent or anamnestic colorectal carcinoma were identified across 2 studies [21, 29]. Histologically, all tumours were adenocarcinomas, including 1 medullary, 2 serrated, and 2 mucinous subtypes. The majority of cases (75.0%, *n* = 9) were located in the proximal colon, whereas 25.0% (*n* = 3) arose in distal segments. These 12 colorectal carcinoma cases occurred in 9 patients, indicating that 3 individuals developed 2 distinct primary colorectal malignancies. Notably, 1 ascending mucinous adenocarcinoma was not only concurrent but also continuous with a mucinous adenocarcinoma of the appendix in the presence of an appendiceal SSL; the authors describe a morphological continuum suggesting progression from the SSL to appendiceal adenocarcinoma [29]. This patient also had a prior history of rectal carcinoma. Two-thirds of affected patients were male (*n* = 6), in contrast to the overall female predominance observed in the broader appendiceal SSL population. The mean age of patients with both appendiceal SSL and colorectal carcinoma was 72.8 years (range: 69–79), approximately one decade higher than the mean age reported for all SSL patients (62.9 years). The demographic data of patients with concurrent or anamnestic colorectal cancer are detailed in Table 4.

**Table 4:** Sex and age of patients with concurrent or anamnestic colorectal cancer

Concurrent/anamnestic neoplasm	Sex	Age	Publication
Cecum adenocarcinoma	M	76	Csonka et al., 2025 [21]
Cecum adenocarcinoma	M	71	Csonka et al., 2025 [21]
Cecum serrated adenocarcinoma	F	76	Csonka et al., 2025 [21]
Ascending colon adenocarcinoma	M^1^	70	Csonka et al., 2025 [21]
Ascending colon adenocarcinoma	M^2^	71	Csonka et al., 2025 [21]
Ascending colon adenocarcinoma	M	71	Csonka et al., 2025 [21]
Ascending colon medullary adenocarcinoma	M	69	Csonka et al., 2025 [21]
Ascending colon mucinous adenocarcinoma	F	79	Csonka et al., 2025 [21]
Ascending colon mucinous adenocarcinoma	F^3^	72	Kinoshita et al., 2014 [29]
Descending colon adenocarcinoma	M^1^	70	Csonka et al., 2025 [21]
Descending colon adenocarcinoma	M^2^	71	Csonka et al., 2025 [21]
Rectum adenocarcinoma	F^3^	72	Kinoshita et al., 2014 [29]

^1,2,3^ – same index indicates the same patient

### Molecular profiling - IHC and molecular genetics

Four studies performed IHC analyses across a total of 32 cases (18.85%). Kinoshita et al. evaluated the expression of MUC2, MUC5AC, MLH1, p53, nuclear β-catenin and MUC6 [29]. Dargent et al. assessed mismatch repair (MMR) proteins (PMS2, MSH2, MLH1, MSH6) as well as the *BRAF* V600E mutation [27]. Chezar and Minoo investigated nuclear β-catenin and Annexin A10 expression in concurrent SSLs and LAMNs [26], while Munari et al. examined MMR proficiency and p53 expression [28]. 

The most frequently observed alteration involved p53 expression: 3 of 19 evaluated cases (15.79%) demonstrated abnormal staining patterns, defined as either strong nuclear staining in more than 30% of neoplastic cells or complete absence of staining in the presence of a positive internal control [28]. Nuclear localisation of β-catenin was identified in 2 cases (15.39%), both of which were associated with concurrent LAMN; in these cases, the LAMN component also exhibited nuclear β-catenin positivity [26]. One case (5.00%) demonstrated apical loss of MLH1 expression along with weak MSH2 staining. This lesion was associated with an appendiceal mucinous adenocarcinoma that showed a similar immunoprofile, except for strong MSH2 expression [29]. Finally, BRAF V600E protein expression was detected in one pediatric case suspicious for SPS [27]. Table 5. summarises the IHC properties of the cases.

**Table 5:** Results of IHC examination of appendiceal SSLs

Examined marker	Examined cases (*n*)	Pathogenic alteration (*n*)	Ratio of examined cases (%)
p53	19	3	15.79%
Nuclear β-catenin	13	2	15.39%
Annexin 10	12	1	8.33%
MLH1	20	1	5.00%
MSH2	20	1*	5.00%
BRAF V600E	1	1	100.00%
MSH6	19	0	0.00%
PMS2	19	0	0.00%

Examined marker	Examined cases (*n*)	Positive reaction (*n*)	Ratio of examined cases (%)
MUC2	1	1	100.00%
MUC5A/C	1	1	100.00%
MUC6	1	0	0.00%

*Abbreviations*: *BRAF* V-raf murine sarcoma viral oncogene homolog B, *MLH1* MutL homolog 1, *MSH2* MutS homolog 2, *MSH6* MutS homolog 6, *MUC2* Mucin-2, *MUC5AC* Mucin-5AC, *MUC6* Mucin-6, *PMS2* Postmeiotic segregation increased 2

* weak staining

Across 5 studies, molecular genetic testing was performed in 58 cases (25.11%), with 41 (70.69%) harbouring at least one pathogenic mutation; 5 cases exhibited multiple pathogenic mutations.

Munari et al. aimed to molecularly characterise serrated lesions of the appendix, including HPs, SSLs, serrated adenomas, and AMNs. Targeted sequencing included the genes *RNF43*,* SMAD4*,* KRAS*,* NRAS*,* BRAF*, and *PIK3CA*. Among the 18 SSLs analysed, *KRAS* mutations were the most frequent, identified in 10 cases (55.56%). *BRAF* mutations were detected in 4 cases (22.22%), while a single case (5.56%) harboured an *RNF43* mutation [28]. 

Dargent et al. reported a rare pediatric case of appendiceal SSLs with suspected SPS. Genetic analysis, supported by IHC, confirmed the presence of a *BRAF* mutation [27]. 

Chezar and Minoo performed a comparative molecular analysis of appendiceal SSLs and LAMNs, examining these lesions both as independent entities and in cases where they co-occurred. The analysis focused on mutations in *BRAF*, *KRAS*, and *GNAS*. Among the 32 cases evaluated, activating *KRAS* mutations were identified in 17 cases (53.1%), while activating mutations in *BRAF* and *GNAS* were each detected in a single case (3.1%). In the subset of cases with concurrent SSL and LAMN (*n* = 12), *KRAS*/*BRAF* genotypes were concordant in 10 cases (83.3%). Identical *KRAS* mutations were present in 6 of 12 cases (50.0%), and the single *BRAF*-mutated case demonstrated the same mutation in both lesions. The presence of identical driver mutations in paired lesions was interpreted by the authors as evidence supporting a potential clonal relationship between SSLs and LAMNs. The principal molecular and immunophenotypic differences between concurrent SSLs and LAMNs involved *GNAS* mutation status and β-catenin expression. As noted above, only one SSL harbored a *GNAS* mutation (1/12, 8.3%), whereas *GNAS* mutations were detected in 4 corresponding LAMNs (4/12, 33.3%). Similarly, aberrant nuclear β-catenin expression was observed in only a minority of SSLs (2/12, 16.7%), while the majority of concurrent LAMNs demonstrated nuclear β-catenin staining (10/12, 83.3%). These findings suggest that, despite sharing common early molecular alterations, SSLs and LAMNs may diverge through the acquisition of additional genetic and signaling pathway abnormalities during neoplastic progression [26]. 

Bauer et al. investigated the genetic landscape of concurrent appendiceal SSLs and various appendiceal adenocarcinomas, including goblet cell adenocarcinomas, using next-generation sequencing (NGS), followed by assessment of the pathogenicity of identified alterations. All cases (*n* = 6; 100%) harboured *KRAS* mutations. Additionally, *GNAS* mutations were identified in 2 cases (33.33%), and a pathogenic *TP53* mutation was detected in a single case (16.67%) [25]. 

**Fig. 10:**
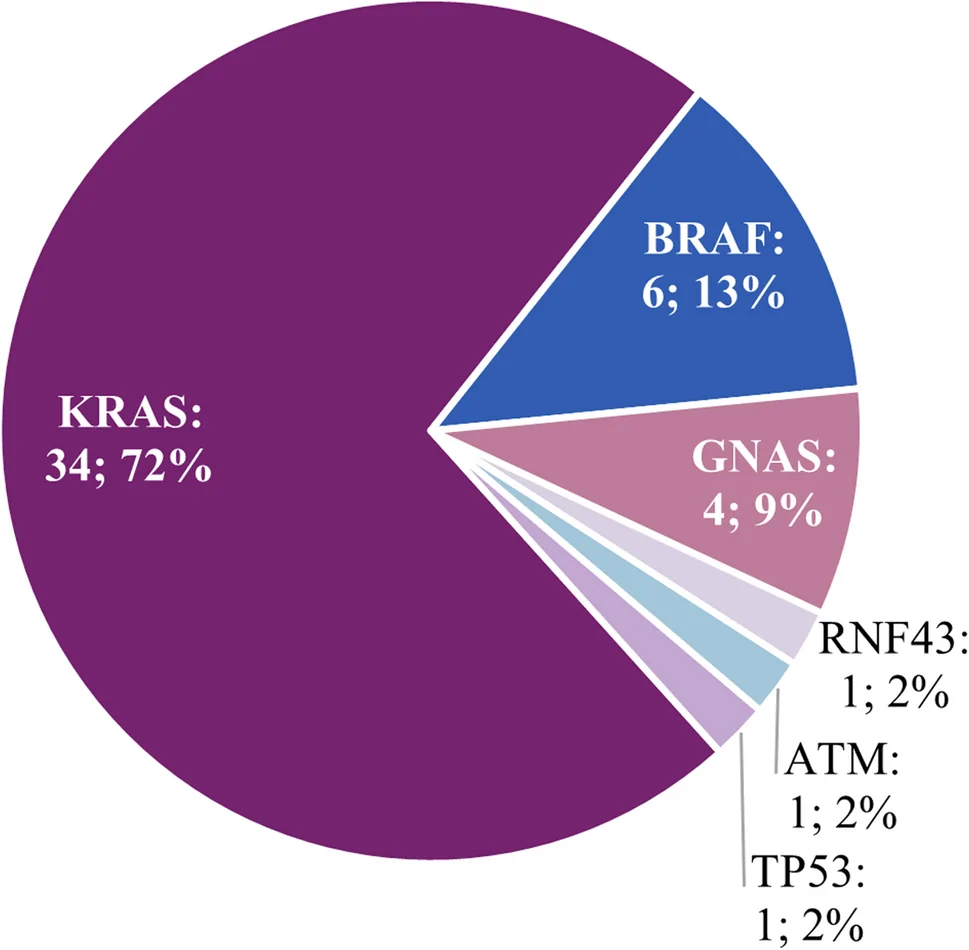
Distribution of gene mutations in appendiceal SSLs. Abbreviations: ATM – Ataxia-telangiectasia mutated; BRAF – V-raf murine sarcoma viral oncogene homolog B; GNAS – Guanine Nucleotide binding protein, Alpha Stimulating activity polypeptide; KRAS – Kirsten rat sarcoma virus oncogene homologue; RNF43 – Ring finger protein 43; TP53 – Tumour protein 53

Mahadik et al. analysed multiple appendiceal neoplasms using NGS, including a single case of an appendiceal SSL associated with signet ring cell carcinoma. This lesion harboured *ATM*,* GNAS*, and *KRAS* mutations, which were concordant with those identified in the associated signet ring cell carcinoma [34]. Table 6 provides insight into the frequency of gene mutations, along with the number of examined cases, and Fig. 10 illustrates the relative ratio of mutations.

**Table 6:** Prevalence of gene mutations in appendiceal SSLs

Examined gene	Examined cases (*n*)	Pathogenic alteration (*n*)	Ratio of examined cases (%)
KRAS	56	34	59.65%
BRAF	57	6	10.53%
GNAS	56	4	7.14%
RNF43	23	1	4.35%
ATM	7	1	14.29%
TP53	7	1	14.29%
NRAS	18	0	0.00%
SMAD	18	0	0.00%
PIK3CA	18	0	0.00%

*Abbreviations*: *ATM* – Ataxia-telangiectasia mutated, *BRAF* V-raf murine sarcoma viral oncogene homolog B, *GNAS* Guanine Nucleotide binding protein, Alpha Stimulating activity polypeptide, *KRAS* Kirsten rat sarcoma virus oncogene homologue, *NRAS* Neuroblastoma RAS viral oncogene homolog, *PIK3CA* Phosphatidylinositol-4,5-bisphosphate 3-kinase, catalytic subunit alpha, *RNF43* Ring finger protein 43, *SMAD4* SMAD family member 4, *TP53* Tumour protein 53

Of the 41 cases involving pathogenic genetic mutations, 16 were associated with a concurrent appendiceal neoplasm. Among the 9 cases of SSL–LAMN co-occurrence, eight demonstrated shared *KRAS* or *BRAF* mutations [26]. In contrast, the 4 cases involving SSL and goblet cell adenocarcinoma showed no shared genetic alterations between the 2 histological entities [25]. Two cases presented with concurrent SSL and appendiceal adenocarcinoma; in both instances, *KRAS* mutations were shared, and a case additionally exhibited a shared *TP53* mutation. Notably, the appendiceal adenocarcinomas in both cases also acquired pathogenic *SMAD4* mutations [25]. In a case of SSL co-occurring with signet ring cell carcinoma, NGS identified 3 shared mutations: *ATM*, *GNAS*, and *KRAS* [34]. 

### An ambiguous entity - clinicopathological features of SSLs at the appendiceal orifice

Among the 231 cases presented above, 34 cases, 14.72% of all cases showcase a special entity among SSLs - lesions located at the appendiceal orifice. Across 12 publications, 34 cases were described, all of which were detected during either screening or diagnostic–therapeutic colonoscopy [8, 9, 12–17, 19, 20, 29, 31]. Demographic data was available in 14 cases (41.18%). Patients with appendiceal orifice SSLs were predominantly female (*n* = 9, 64.29%). The mean age at diagnosis was 59.3 years (range: 36–75 years). Exact lesion size was available in 10 cases (29.41%), the mean lesion size was 15.2 mm, ranging from 8 mm to a maximum of 28 mm. In the 11 cases with data regarding the presence of dysplasia, 2 (18.18%) SSL-Ds were diagnosed.

Most patients were asymptomatic (12/13, 92.31%), a single patient (7.7%) presented with chronic constipation. In three cases that were discussed above, appendiceal orifice growths were detected post-appendectomy, either directly or decades after surgery [14, 15, 20]. Three patients had a history of diagnosed appendiceal orifice SSLs, two had undergone repeated biopsies, always revealing SSL upon histological examination [14, 31]. In one case initial resection of the lesion was partial, the patient later underwent laparoscopic appendectomy with cecal cuff resection. Furthermore Harris et al. suggests the above mentioned surgical approach to be the standard therapeutic pathway for SSLs located at the appendiceal orifice. [12]

Among the reported appendiceal orifice SSL cases two have been associated with other appendiceal neoplasms. Kinoshita et al. reported a gradual transition from the appendiceal orifice SSL to appendix mucinous adenocarcinoma. [29] Wurtz et al. have described concurrent SSL and appendiceal NET. [31] None of the reported cases have been associated with colorectal neoplasms.

None of the above mentioned publications have carried out genetic testing. In one instance IHC has been carried out with various markers (MUC2, MUC5AC, MUC6, MLH1, MSH2, p53), which revealed a shared loss of MHL1 expression in the co-occurring SSL and mucinous adenocarcinoma [29]. Further investigation regarding the origin of the lesions has not been described in the studies.

## Discussion

Appendiceal SSLs are benign appendiceal neoplasms characterised by serrations extending to the base of the intestinal crypts, along with basal crypt dilation and architectural distortion, including horizontal crypt growth above the lamina muscularis mucosae. The terminology was formally updated in the 2019 WHO Classification [2]; prior to this, the term SSP/A was commonly used. The earliest use of the term “appendiceal SSL” in the scientific literature dates to 2014. Although it appeared in a case report predating the official nomenclature change, the histological features and definitions presented are consistent with the entity now recognised as appendiceal SSL [29]. 

The transition from the previously used SSP/A terminology to SSL has been gradual; however, the concurrent use of both terms complicates comparisons across studies, as the change encompassed not only nomenclature but also revised diagnostic criteria. This has led to a degree of diagnostic drift, rendering epidemiological comparisons between SSP/As and SSLs less reliable. A review published in 2023, drawing on studies from 2015 to 2018, reported a collective prevalence of SSLs and adenomas of 1.11‰ in appendectomy specimens [35]. Núñez-Rocha et al. reported a somewhat higher prevalence of SSP/As at 4.01‰ per appendectomy, still applying the earlier nomenclature [36]. In comparison, a more recent estimate of 5.22‰ might suggest an apparent increase in incidence; however, such an interpretation is likely confounded by differences in diagnostic frameworks rather than reflecting a true rise in occurrence [21]. 

Current data indicate variability in the prevalence of appendiceal SSLs across different populations, although these lesions remain rare in all settings. Among patients undergoing appendectomy for acute appendicitis, the prevalence of SSLs has been reported at 5.22‰; however, in cohorts restricted to individuals over 40 years of age, this figure increases approximately fourfold to 21.51‰ [21, 22]. In populations affected by SPS, the prevalence rises more than 17-fold, reaching 87.72‰ [24]. The age-related increase in prevalence is consistent with the observed age distribution of appendiceal SSLs. The highest incidence occurs in patients aged 61–80 years, while fewer than one-quarter of cases are identified in individuals under 40 years of age. Additionally, over 60% of affected patients are female. Overall, increasing age and the presence of SPS suggests association with a higher prevalence of appendiceal SSLs [22, 24]. 

Most appendiceal SSLs are macroscopically inconspicuous and are typically identified only upon histological examination. Reporting on lesion location is notably imbalanced, as most available data derive from endoscopic procedures. This likely contributes to the apparent predominance of proximally located lesions, particularly those visible at the appendiceal orifice. In contrast, earlier studies based on surgical specimens of SSP/A suggest a tendency toward more distally located lesions [37]. Dysplasia is not a predominant feature, with only 28.38% of cases demonstrating dysplastic components. Overall, most lesions are detected either incidentally during routine colonoscopy – primarily proximal lesions – or through histopathological evaluation of specimens obtained from surgically treated appendicitis cases [8–22, 26, 27, 33].

SSLs may arise not only in the vermiform appendix but also throughout the colorectum. Despite their shared origin within the large intestine, some authors propose that colorectal and appendiceal SSLs should not be considered a homogeneous entity distinguished solely by anatomical location, as important genetic and prognostic differences exist between them. Pai et al. advocate in their 2014 publication, that serrated lesions of the appendix may look identical to their colonic counterparts, however this morphologic similarity is misleading and applying diagnostic criteria developed for colonic lesions may not be entirely appropriate. The authors argue that the major discordance in genetic mutations between appendiceal and colonic lesions strongly suggests distinction between them [37]. Their argument is backed by a more recent publication that applies current diagnostic criteria regarding appendiceal and colonic SSLs. Chezar and Minoo in 2022 have found that a previously identified serrated pathway marker, Annexin 10 shows significantly different expression in appendiceal and colonic SSLs. Only a minority (1/12, 8.3%) of appendiceal lesions showed Annexin 10 positivity with IHC, however all examined colonic lesions (14/14, 100%) stained positively for the marker. In the authors’ interpretations this finding strengthens the argument suggesting different biological pathways for SSLs arising in the appendix and the colon [27]. However, apart from these 2 publications, during our research, no further studies were identified that would have provided further evidence regarding the issue.

The mean age at diagnosis for colonic SSLs is approximately 63 years, closely mirroring that reported for appendiceal SSLs. However, while colonic SSLs are generally described as having no significant sex predilection, appendiceal lesions demonstrate a female predominance [6]. In terms of prevalence, colonic SSLs are considerably more common, occurring in an estimated 20–80‰ of the general population [38]. Exact comparison between the prevalence of appendiceal and colonic SSLs is not possible as all appendiceal SSL prevalence data describes special patient populations, rather than a general population. Dysplasia appears less frequently in colonic SSLs, with only 1–14% of cases progressing to SSL-D [39]. Lesion size is a recognised risk factor for both the presence of dysplasia and progression to colorectal carcinoma [39]. A direct comparison with appendiceal SSLs is limited, however, due to insufficient overlapping data on lesion size and dysplastic status in reported cases. Colonic SSLs are well-established premalignant lesions, with approximately 15–40% of sporadic colorectal carcinomas arising via the serrated pathway [6, 39]. The 10-year risk of progression to colorectal carcinoma is estimated at 3.2% for SSLs and 4.4% for SSL-D [38]. Failure to detect these lesions during colonoscopy has been implicated in the development of post-colonoscopy colorectal cancer [39]. Although appendiceal SSLs may also be missed during endoscopic evaluation, it would be inappropriate to infer a similarly high risk of post-endoscopic appendiceal carcinoma. Available evidence suggests a more indolent course, supported by reports of prolonged non-progression, including a documented case with 12 years of follow-up [24, 31]. Genetically, the two entities also diverge. Activating *BRAF* mutations are present in approximately 75–90% of colonic SSLs, whereas they are identified in only about 10.53% of appendiceal cases [38]. Conversely, *KRAS* mutations are less common in colonic lesions but occur in more than half of appendiceal SSLs [1]. In the colorectum, progression from SSL to SSL-D is strongly associated with loss of *MLH1* expression; however, current data are insufficient to determine whether this mechanism plays a comparable role in appendiceal lesions [6]. 

A liminal entity right at the anatomical margin of the vermiform appendix is the appendiceal orifice SSL. In the cases reviewed, a substantial proportion of appendiceal SSLs were located at the appendiceal orifice or extended into the cecum [8, 9, 12–17, 19, 20, 29, 31]. There is some level of uncertainty regarding the origin of these lesions - whether they arose in the appendix and spread to the appendiceal orifice, potentially to the cecum or vice versa. Given the differing risk profiles of colonic and appendiceal SSLs, distinguishing between these entities is clinically relevant. The studies presenting appendiceal orifice SSL cases mainly present demographic and some histologic data, therefore, meaningful conclusions cannot be drawn. The mean age at diagnosis is similar in all three groups (appendiceal SSL: 62.9 years, appendiceal orifice SSL: 59.3 years, colonic SSL: 63 years) [6, 8–10, 12, 14–21, 23, 26, 27, 29, 31, 32]. Appendiceal orifice SSLs showed female predominance (64.29%), a higher rate of dysplasia (18.18%) similarly to appendiceal SSLs [8–10, 12, 14–21, 23, 26, 27, 29–32]. Most likely due to the mainly exploratory, screening endoscopies, the majority of appendiceal orifice lesions were asymptomatic (92.31%), whereas appendiceal SSLs were symptomatic in over 40% of the reported cases [8–10, 12, 14, 16–24, 27, 29, 31, 32]. The lack of genetic and natural disease progression data prevents meaningful comparison, to test the potential similarities or differences in mutational pathways among these entities. Accurate differentiation could have important implications for surveillance strategies and risk assessment. However, to date, no studies have established reliable criteria for determining the site of origin in SSLs with ambiguous anatomical localisation.

SPS is primarily a colonic disorder characterised by the presence of numerous serrated polyps, including HPs, SSLs, and TSAs [38, 39]. The condition affects approximately 1‰ of the general population and is associated with a roughly tenfold increased risk of developing colorectal carcinoma [39]. The relatively high prevalence of appendiceal SSLs observed in this population suggests that SPS may also predispose to the development of appendiceal lesions. Molecular profiling has demonstrated that a substantial proportion of appendiceal SSLs harbour genetic alterations, most commonly activating *KRAS* mutations. In contrast to colonic SSLs, *BRAF* mutations are comparatively infrequent in appendiceal lesions. These findings support the concept that appendiceal SSLs follow a partially distinct molecular pathway relative to their colonic counterparts. This observation is consistent with earlier findings by Pai et al., who reported that appendiceal SSP/As differ from colonic lesions in their genetic profile, with *KRAS* mutations predominating over *BRAF* alterations [37]. 

The prognosis and premalignant potential of appendiceal SSLs remain uncertain. One case report described a patient with an SSL persisting for over a decade without evidence of progression or dysplasia [31]. In contrast, another report documented continuous recurrence and gradual enlargement of an SSL-D in the appendiceal stump over a 5-year period [14]. Given the scarcity of longitudinally followed and repeatedly sampled cases, no definitive conclusions can be drawn. Furthermore, the lack of detailed case-level data in many studies precluded meaningful comparison of mutation profiles between SSLs and SSL-Ds. Frequent co-occurrence of SSLs and LAMNs has been reported, however publication bias likely swayed the frequency of concurrence. Shared genetic alterations – most notably *KRAS* mutations – along with the presence of activated Wnt signalling in a substantially higher proportion of LAMNs (83.33%) compared to SSLs (16.67%), introduce the question of a potential biological relationship between these entities [26]. Although further evidence is needed, available data merely circumscribes the topic of discussion, there is a possibility that some LAMNs may arise from pre-existing SSLs, a concept analogous to the progression of colonic SSLs to invasive carcinoma, where *KRAS* acts as an early driver mutation and increased Wnt signalling reflects a later progression event [40]. Additional information for a potential progression pathway is provided by a reported case of concurrent SSL and appendiceal mucinous adenocarcinoma, which demonstrated a gradual micromorphological transition between the lesions alongside shared immunohistochemical features, including *MLH1* deficiency [29]. Similarly, 2 further cases of concurrent SSL and adenocarcinoma showed shared *KRAS* mutations, with one also harbouring a *TP53* alteration; both adenocarcinomas additionally exhibited *SMAD4* mutations [25]. In contrast, molecular analyses available in four cases make it seem that SSLs and goblet cell adenocarcinomas are likely unrelated, representing distinct neoplastic lineages [25]. 

If a sequential pathway exists in which appendiceal SSLs progress to dysplasia, LAMN, or adenocarcinoma through stepwise acquisition of genetic alterations, it is likely an uncommon event. Publication bias may further exaggerate the perceived frequency of these associations, as unusual co-occurrences and atypical histological presentations are more likely to be reported. Consequently, the true prevalence of SSL-associated neoplasms remains unknown. Robust confirmation or refutation of such progression would require additional cases with comprehensive genetic profiling.

Appendiceal SSLs represent an elusive entity, with approximately 90% of lesions undetected prior to histological evaluation, according to the reviewed literature. Consequently, careful histopathological examination of appendectomy specimens remains a critical diagnostic safeguard. The low detection rate likely reflects the absence of macroscopic changes on the serosal surface, as SSLs are confined to the tunica mucosa. Endoscopic visualisation offers improved detection, but standard colonoscopy is largely limited to identifying lesions at the appendiceal orifice based on historical data it is likely that a significant proportion of SSLs arelocated in the middle or distal third of the appendix. Four case reports have described the use of a sub-scope or cholangioscope inserted into the appendiceal lumen during colonoscopy to directly visualise the mucosa [10, 17–19]. While this approach is not part of routine clinical practice, it may represent a potential strategy for enhancing mucosal visualisation in selected high-risk patients.

Nearly half of appendiceal SSLs are identified incidentally during surgical treatment for acute appendicitis, indicating that therapeutic decisions in this context directly affect the detection of neoplastic lesions within the appendix. Di Saverio et al., in their recommendations for the management of acute appendicitis, highlight that the recurrence of cases following non-operative management remains a continuous concern in clinical practice [41]. In parallel, it has been reported that neoplastic lesions, including appendiceal SSP/A, were identified in 20% of patients over 40 presenting with periappendiceal abscesses [42]. While non-operative management is generally limited to uncomplicated acute appendicitis, findings from Jolly et al. indicate that the higher prevalence of SSLs in patients over 40 is not restricted to complicated cases [22]. These observations suggest that the age-related increase in SSL prevalence extends beyond patients with complicated appendicitis. Given the uncertain prognosis and progression rates of appendiceal SSLs, these data raise important considerations regarding conservative management. In patients over 40 or those with SPS, cautious application of non-operative strategies may be warranted. When surgical intervention is not immediately indicated, an interval appendectomy could be considered to mitigate potential risk [43]. 

This study has several limitations. Although updated terminological recommendations were introduced 7 years ago, published data on appendiceal SSLs remain limited. The evolution of serrated lesion nomenclature over time creates uncertainty when comparing studies from different diagnostic eras. A substantial proportion of the available literature consists of case reports and case series, which provide limited information regarding the clinicopathological characteristics of SSLs, and introduces substantial risks of selection bias. Moreover, such study designs are inherently subject to publication bias, as atypical or unusual presentations are more likely to be reported, potentially leading to underrepresentation of more typical morphological and clinical features. This may have influenced the reported frequencies of concurrent appendiceal neoplasms and appendiceal orifice SSLs, thereby obscuring their true distribution and characteristics. The inclusion of studies published before the adoption of contemporary WHO criteria represents a potential source of diagnostic heterogeneity, as historical cases could only be aligned with the current concept of SSLs based on published pathological descriptions, with independent histological re-evaluation. Aware of these limitations the study strictly refrained from using historical cases during collection of clinicopathological data for appendiceal SSLs, that apply a previous diagnostic framework. On the other hand, in the discussion section some historical data has been carefully utilized for solely illustrative purposes.

Comparative analysis across studies was further constrained by heterogeneity in study design and variability in reported clinicopathological parameters. In several instances, data were presented in aggregate form, precluding the extraction of individual case-level details and limiting inclusion in the collated database. The inconsistent reporting of key parameters, such as lesion size, anatomical location, and the presence of dysplasia, may also affect the robustness of the interpretations. Similar limitations apply to molecular profiling, as among 231 cases, different genetic markers were assessed in only 1–56 cases, resulting in substantial variability in available molecular data. Furthermore, information on potential risk factors, including lifestyle factors such as tobacco smoking and alcohol consumption, as well as comorbidities such as obesity and other chronic diseases, was generally lacking.

The descriptive findings should be interpreted with caution, given the likely overrepresentation of unusual, advanced, or neoplasia-associated cases in the published literature. The principal conclusion of the review is that appendiceal SSLs represent an incompletely characterized lesion category for which substantial clinicopathological and molecular uncertainties remain, underscoring the need for larger, systematically studied and multicenter cohorts and prospective registries with standardized diagnostic criteria and long-term follow-up, to further elucidate the epidemiological, pathological, and molecular characteristics of appendiceal sessile serrated lesions.

## Conclusion

Appendiceal SSLs represent a rare and incompletely characterised entity within the spectrum of serrated appendiceal neoplasia. Although their histomorphological criteria are now well-defined in the WHO 5TH edition classification, the historical coexistence of SSP/A and SSL terminology has resulted in persistent diagnostic heterogeneity, limiting the comparability of published data and contributing to epidemiological uncertainty. Current evidence indicates that appendiceal SSLs are uncommon lesions with a variable but generally low reported prevalence, which increases with age and is particularly elevated in patients with SPS, suggesting a potential predispositional relationship.

Clinically, appendiceal SSLs are most often incidental findings, either detected during colonoscopic evaluation of the appendiceal orifice or identified histologically in appendectomy specimens. The majority of lesions are macroscopically inconspicuous, and a substantial proportion are diagnosed in the context of acute appendicitis. Dysplasia is present in a minority of cases, and long-term clinical behaviour remains insufficiently defined, with limited longitudinal data suggesting both indolent and potentially progressive courses.

Molecularly, appendiceal SSLs appear to differ from their colorectal counterparts, most notably by a lower frequency of *BRAF* mutations and a relative predominance of *KRAS* alterations, supporting the hypothesis of partially distinct neoplastic pathways. Emerging data also suggest potential molecular overlap between appendiceal SSLs and mucinous neoplasms, including LAMNs, raising the possibility of shared early driver events; however, evidence for a definitive stepwise progression remains limited and inconclusive.

Overall, the current literature is constrained by small sample sizes, heterogeneous study designs, inconsistent reporting, and substantial publication bias, particularly toward unusual or concurrent neoplastic findings. These limitations preclude definitive conclusions regarding the true prevalence, natural history, and malignant potential of appendiceal SSLs. Further large-scale, systematically characterised, and longitudinally followed cohorts with comprehensive molecular profiling are required to clarify their biological behaviour and clinical significance.

## Data Availability

This study is based on previously published data. All data sources are cited within the article. No new data were generated.

## Abbreviations

AAp.: Acute appendicitis
AMN: Appendiceal mucinous neoplasm
Asymp.: Asymptomatic
ATM: Ataxia-telangiectasia mutated
BRAF: V-raf murine sarcoma viral oncogene homolog B
Chr.: Chronic
DOI: Digital Object Identifier
EMR: Endoscopic mucosal resection
GNAS: Guanine Nucleotide-binding protein, Alpha Stimulating activity polypeptide
HP: Hyperplastic polyp
IHC: Immunohistochemistry
KRAS: Kirsten rat sarcoma virus oncogene homolog
LAMN: Low-grade appendiceal mucinous neoplasm
MLH1: MutL homolog 1
MMR: Mismatch repair
MSH2: MutS homolog 2
MSH6: MutS homolog 6
MUC2: Mucin-2
MUC5AC: Mucin-5AC
MUC6: Mucin-6
NA: Not applicable
NET: Neuroendocrine tumour
NGS: Next-generation sequencing
NRAS: Neuroblastoma RAS viral oncogene homolog
p53: Protein 53
PIK3CA: Phosphatidylinositol-4,5-bisphosphate 3-kinase, catalytic subunit alpha
PMS2: Postmeiotic segregation increased 2
PRISMA: Preferred Reporting Items for Systematic Reviews and Meta-Analyses
PROSPERO: Prospective Register of Systematic Reviews
RLQ: Right lower quadrant
RNF43: Ring finger protein 43
SMAD4: SMAD family member 4
SPS: Serrated polyposis syndrome
SSL: Sessile serrated lesion
SSL-D: Sessile serrated lesion with dysplasia
SSP/A: Sessile serrated polyp/adenoma
TP53: Tumour protein 53
TSA: Traditional serrated adenoma
WHO: World Health Organization
